# Effect of lidocaine on acute pain after modified radical mastectomy: a secondary analysis of a randomized trial

**DOI:** 10.3389/fmed.2026.1832526

**Published:** 2026-05-26

**Authors:** Jiao Liu, Wenjuan Zhang, Xiaohui Li, Danting Jia, Zhixia Bai, Xuexin Chen

**Affiliations:** 1 Department of Anesthesia and Perioperative Medicine, Cancer Hospital, General Hospital of Ningxia Medical University, Yinchuan, Ningxia, China; 2 Department of Anesthesiology, People's Hospital of Ningxia Hui Autonomous Region, Yinchuan, Ningxia, China

**Keywords:** acute postoperative pain, anesthesia maintenance, inflammatory response, intravenous lidocaine, modified radical mastectomy

## Abstract

**Background:**

Postoperative pain is a common concern for patients undergoing modified radical mastectomy (MRM) for breast cancer. Intravenous lidocaine may alleviate acute postsurgical pain. This study aimed to evaluate the analgesic effect of lidocaine in patients receiving sevoflurane or propofol maintenance anesthesia.

**Methods:**

This study is a secondary analysis of a randomized controlled trial evaluating the effect of lidocaine on postoperative outcomes. One hundred patients scheduled for MRM were randomized into four groups (*n* = 25 per group): sevoflurane (S), sevoflurane plus lidocaine (SL), propofol (P), or propofol plus lidocaine (PL). The primary outcome was the area under the curve (AUC) of the numerical rating scale (NRS) score at rest and during movement within 24 h postoperatively, while secondary outcomes included resting and active NRS within 24 h postoperatively, changes in early inflammatory markers (IL-6, IL-1 β, TNF–α, NF-κ B), consumption of anesthetics and analgesics, adverse events, and patient satisfaction.

**Results:**

Compared with their respective control groups, lidocaine infusion in Groups SL and PL significantly reduced the resting and active AUC for NRS score at 24 h postoperatively (S vs. SL, *P* < 0.001; P vs. PL, *P* < 0.001) and yielded lower resting and active NRS scores at 3, 6, and 12 h after surgery (all *P* < 0.001). No significant intergroup differences were observed in perioperative immune cell counts. However, postoperative serum levels of IL-6, IL-1 <, TNF-α, and NF-κB activity were significantly lower in the lidocaine groups (SL and PL) than in the controls (S and P). The consumption of anesthetics and analgesics, incidence of adverse events, and patient satisfaction within 24 h were comparable among the four groups.

**Conclusions:**

Intraoperative intravenous lidocaine infusion reduced acute postoperative pain, attenuated the early systemic inflammatory response in MRM patients. These benefits were independent of the maintenance anesthetic used (sevoflurane vs. propofol). However, given the multiple confounding factors that may affect the results of this study due to its design, further randomized controlled trials are required to confirm causality and assess long-term clinical and mechanistic outcomes.

**Clinical trial registration:**

ChiCTR2300068563 (registered February 23, 2023).

## 1 Introduction

Breast cancer remains the most commonly diagnosed malignancy among women worldwide (1), and surgery remains an indispensable component of its multimodal treatment (2). Despite the use of multimodal analgesia, postoperative pain following breast cancer surgery continues to pose a significant clinical challenge, affecting 30–50% of patients (3). Inadequately controlled acute pain impairs early recovery, increases opioid consumption, prolongs hospital stay, and may contribute to the development of chronic postsurgical pain, a condition affecting 10–50% of patients and diminishing long-term quality of life (4, 5). Therefore, effective perioperative pain management strategies are critical in this population.

The perioperative period is crucial for optimizing pain control. Surgical stress enhances the release of pro-inflammatory cytokines such as interleukin-1β (IL-1β), interleukin-6 (IL-6), and tumor necrosis factor-alpha (TNF-α), which influence postoperative pain through neuronal pathway activation and sensitization (6). The selection of anesthetic techniques and agents is central to perioperative pain management. Current practice increasingly emphasizes multimodal analgesia that combines regional anesthesia with systemic agents including opioids, nonsteroidal anti-inflammatory drugs (NSAIDs), and local anesthetics (7). However, the implementation of regional analgesic techniques is often limited by equipment availability and technical expertise, and carries risks such as pneumothorax and hematoma (8). Meanwhile, opioid use is constrained by adverse effects including nausea, vomiting, respiratory depression, and potential for dependence (9). Consequently, identifying safe, effective, and easily applicable adjunct analgesic methods to optimize multimodal analgesia, reduce acute pain, and prevent chronic pain development has become a key research priority in perioperative anesthesia management.

Lidocaine is a commonly used amide local anesthetic (10). Recent studies have demonstrated that intravenous lidocaine exerts anti-inflammatory effects, modulates ectopic neuronal discharge, and inhibits central sensitization, mechanisms that may contribute to beneficial modulation of postoperative pain (11). Compared with regional blockade, intravenous administration is technically simple, compatible with continuous infusion during general anesthesia, and offers favorable clinical feasibility (12). Intravenous lidocaine has also been shown to reduce opioid consumption and decrease pain scores following abdominal and thoracic surgeries (13). However, some studies suggest that its efficacy for postoperative pain control in cancer surgery remains uncertain (14). Moreover, whether the analgesic effect of lidocaine as an adjunct in cancer surgery is influenced by the choice of maintenance anesthetic, propofol or sevoflurane, remains unclear. Propofol and sevoflurane differ in their effects on hepatic perfusion and drug-metabolizing enzymes, which may alter lidocaine clearance and bioavailability (15). Furthermore, clinical studies systematically comparing the effects of these two anesthetic agents on acute postoperative pain in cancer populations are limited and have yielded inconsistent results (16). Some studies indicate that propofol anesthesia reduces postoperative pain scores and opioid consumption compared with sevoflurane in patients undergoing cancer surgery (17, 18), whereas others report conflicting findings (19, 20). Therefore, we hypothesized that intravenous lidocaine reduces acute postoperative pain in breast cancer surgery, and that this effect differs between patients receiving propofol versus sevoflurane maintenance anesthesia.

We conducted an in-depth analysis of data from a previous clinical trial to systematically evaluate the effects of intraoperative intravenous lidocaine on acute postoperative pain, early inflammatory markers, and opioid consumption in patients undergoing modified radical mastectomy (MRM) under propofol or sevoflurane maintenance anesthesia. This study aims to provide clinical evidence for optimizing perioperative analgesic strategies and to further clarify the therapeutic value of intravenous lidocaine in this setting.

## 2 Methods

### 2.1 Study design and participants

This secondary analysis study is based on a randomized controlled trial, which was prospectively registered at www.chictr.org.cn (ChiCTR2300068563) on Feb 23, 2023. The original protocol prespecified neutrophil extracellular traps (NETs) markers as the primary endpoint and NRS-measured postoperative pain at discrete time points (e.g., preoperative, 0 h, 3 h, 6 h, 12 h, 24 h) as a secondary endpoint (21). The experimental protocol was approved by the Ethics Committee of Ningxia Medical University General Hospital (KYLL-2023-0045) and conducted in accordance with the Declaration of Helsinki. The study ran from Mar 01, 2023 to Oct 01, 2023. We performed this study in accordance with Consolidated Standards of Reporting Trials (CONSORT) guidelines. The first patient was enrolled on Mar 01, 2023. Written informed consent was obtained from all participating patients after detailed explanation of the study procedures, potential risks, and benefits. The current analysis includes fewer participants than the original trial because we required complete NRS recordings at all time points for AUC calculation; participants with any missing pain assessment were excluded. All other inclusion/exclusion criteria, intervention protocol, and measurement methods were identical to the original RCT, minimizing selection bias.

We included patients who met the following criteria: (1) Adult female patients aged 18–70 years; (2) American Society of Anesthesiologists (ASA) status I-III; (3) BMI 18–27 kg/m^2^ (Avoids extremes that may alter drug distribution or perioperative complications); (4) Scheduled for MRM. Exclusion Criteria included: (1) Refusal or inability to provide informed consent; (2) Allergy or contraindication to study agents; (3) Chronic consumption of opioids, other analgesics or anti-inflammatory drugs.

Randomization and Blinding: Eligible patients were randomized using a computer-generated sequence with sealed opaque envelopes (1:1:1:1): Group S: Sevoflurane-anesthesia + Placebo (Normal saline) Infusion; Group SL: Sevoflurane-anesthesia + Lidocaine Infusion; Group P: Propofol-TIVA + Placebo (Normal saline) Infusion; Group PL: Propofol-TIVA + Lidocaine Infusion. Patients, surgeons, anesthesiologists involved in postoperative care, data collectors, and outcome assessors were blinded to group assignment and the content of the infusion. The study infusion was prepared by an independent anesthesiologist not involved in the patient's care or data collection.

### 2.2 Anesthesia and perioperative care

Standard preoperative fasting guidelines were followed, and no premedication was administered. Standard monitoring (electrocardiography, non-invasive blood pressure, pulse oximetry, and bispectral index) was applied. Anesthesia was induced with intravenous midazolam (0.05 mg/kg), sufentanil (0.3 μg/kg), etomidate (0.3 mg/kg), and rocuronium (0.6 mg/kg) to facilitate laryngeal mask airway (LMA) insertion.

Anesthesia was maintained with either sevoflurane (0.7–1.3 MAC) in Groups S and SL or a propofol infusion titrated to maintain a BIS value between 40 and 60 in Groups P and PL. All patients received a remifentanil infusion 0.2–0.4 μg/(kg·min) to provide continuous intraoperative analgesia. Volume-controlled ventilation with an air-oxygen mixture (FiO_2_ 0.5) was adjusted to maintain normocapnia (EtCO_2_ 35–45 mmHg). Lactated Ringer's solution was infused at 4–6 ml/(kg·h). Blood loss was replaced with crystalloids or colloids as clinically indicated. Hypotension unresponsive to fluid administration (MAP decrease >20% from baseline or MAP <60 mmHg) was treated with ephedrine or phenylephrine. Atropine was given when the heart rate was lower than 50 bpm. Additional rocuronium was administered based on train-of-four (TOF) monitoring.

In the lidocaine groups (SL, PL), patients received an intravenous bolus of 1.5 mg/kg lidocaine (1%) 10 min before induction, followed by a continuous infusion at 2 mg/(kg·h) until the end of surgery. Patients in the control groups (S, P) received equal volumes of normal saline at the same time points. The bolus and infusion doses were selected based on a previously published regimen shown to be effective and safe (22). A lipid emulsion was immediately available to treat any signs of local anesthetic systemic toxicity (LAST).

All surgeries were performed by experienced breast surgeons using standardized techniques. No patients in this study received local anesthetic infiltration at the incision site. The duration of surgery (from skin incision to closure) and anesthesia (from induction to extubation) were recorded for each patient. At the end of surgery, neuromuscular blockade was reversed with neostigmine (0.04 mg/kg) and atropine (0.02 mg/kg). Extubation was performed once standard criteria were met, and patients were transferred to the post-anesthesia care unit (PACU).

Postoperative pain was assessed regularly in the PACU and on the ward using an 11-point numeric rating scale (NRS) (0 = no pain, 10 = worst imaginable pain). Rescue analgesia, consisting of intramuscular ketorolac (30 mg) for an NRS score ≥4, followed by oral paracetamol (1 g every 8 h) if needed, was administered by blinded nursing staff.

### 2.3 Outcomes

The primary outcome was the area under the curve (AUC) of the NRS score at rest and during movement within 24 h postoperatively. The AUC of the resting NRS score over the first 24 h after surgery was defined as the area under the curve formed by plotting time (0–24 h) on the x-axis and the resting NRS score on the y–axis. The AUC of the NRS score during movement (Active AUC) was defined similarly. These two metrics reflect the total burden of pain intensity and duration under resting and movement conditions, respectively. Both AUCs were calculated using the trapezoidal method: resting and movement NRS scores (0–10) were recorded simultaneously at multiple predefined time points (e.g., 0, 3, 6, 12, and 24 h postoperatively). For the resting AUC, the trapezoidal area between consecutive time points was computed as [(t_2_-t1) × (NRS1+NRS_2_)/2] and then summed in chronological order. The Active AUC was calculated in the same manner using the movement NRS scores. The two AUCs were derived independently and were not combined into a total area. The units for both the resting and movement AUCs are “NRS·hour” (NRS·h). These metrics can be used separately to evaluate the overall pain control efficacy of an analgesic regimen at rest and during movement (23).

Secondary outcomes included postoperative pain scores assessed using the NRS at rest and during movement, recorded 10 min before surgery and at 0, 3, 6, 12, and 24 h postoperatively. Inflammatory and immune parameters were measured from blood samples collected from the non-surgical arm 10 min before surgery and 3 h after surgery. These included neutrophils, lymphocytes, and platelets counts, as well as derived inflammatory indices such as the neutrophil-to-lymphocyte ratio (NLR), lymphocyte-to-monocyte ratio (LMR), platelet-to-lymphocyte ratio (PLR). Levels of inflammatory cytokines, including IL-6, IL-1β, TNF-α, and nuclear factor kappa-B (NF-κB), were quantified by enzyme-linked immunosorbent assay (ELISA) from the same blood samples. Perioperative consumption of anesthetics and analgesics, including sufentanil, propofol, remifentanil, lidocaine, ephedrine, ketorolac, and was recorded. Adverse events and patient satisfaction were also documented. Patient satisfaction was assessed at 24 h postoperatively using an 11-point Likert scale ranging from 0 (completely dissatisfied) to 10 (completely satisfied) (24).

Adverse events monitored and recorded included PONV, respiratory depression (respiratory rate <8 breaths per minute or SpO_2_ <90% on room air), hypotension (MAP <60 mmHg or decrease > 20% from baseline requiring intervention), hypertension (MAP > 110 mmHg or increase > 20% requiring intervention), arrhythmias (e.g., bradycardia <50 bpm, tachycardia > 120 bpm, ectopic beats), neurological symptoms (perioral numbness, tinnitus, dizziness, visual disturbances, muscle twitching), signs of LAST, and allergic reactions.

### 2.4 Sample size calculation and statistical analysis

Sample size was calculated using PASS software. Based on our pilot study involving 12 patients, the median and interquartile range (IQR) of the AUC for the 24–h postoperative NRS pain score during movement were 74.3 (65.4, 82.6) in the S group and 60.6 (50.3, 68.5) in the SL group. Sample size estimation was performed using an analysis of the Kruskal-Wallis test with Bonferroni correction for multiple comparisons. With a two-sided significance level of 0.00833 after correction and a desired power of 0.8, the required total sample size was 80 patients (20 per group). Assuming a dropout rate of 20%, the sample size was increased to 100 patients (25 per group) to ensure adequate statistical power.

Statistical analyses were performed using SPSS (version 27.0, IBM Corp.) and Prism (version 10.0, GraphPad Software). Data normality was assessed using the Shapiro-Wilk test and Q-Q plots. Normally distributed continuous data are presented as mean ± standard deviation (SD) and were analyzed using one-way ANOVA. Non-normally distributed or ordinal data are presented as median (IQR). For non-normally distributed repeated-measures data, the Friedman test was used; for multi-group comparisons, the Kruskal-Wallis test was used; for two-group comparisons, the Wilcoxon rank-sum test was used. The Bonferroni method was used to adjust for multiple comparisons, and the significance threshold was set at α = 0.05/6 ≈ 0.0083. After adjustment, a *P*-value <0.0083 was considered to indicate a statistically significant difference.

## 3 Results

### 3.1 Demographic and perioperative characteristics

**Figure 1:**
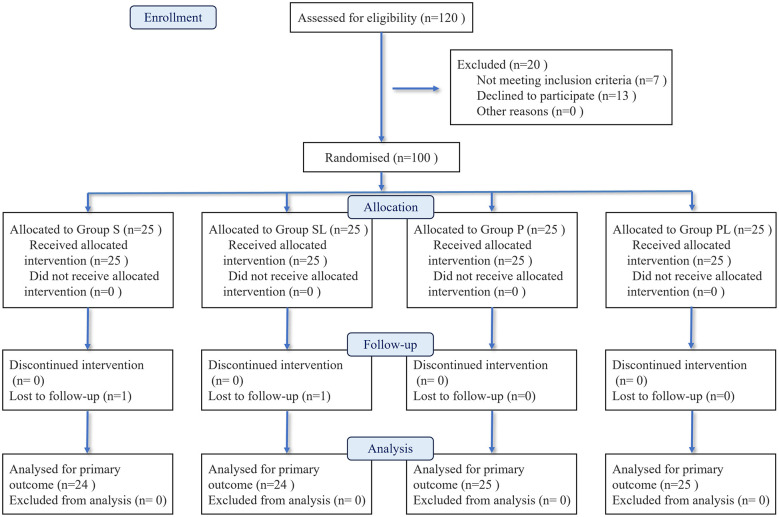
Consort flow diagram.

The study flowchart is shown in Figure 1. From February 2023 to August 2023, 120 patients were assessed for eligibility. After excluding 20 patients, we randomized the remaining 100 into four groups, 25 patients each group. Two patients were excluded after randomization and a total of 98 subjects was included in the final population. As shown in Table 1, the four groups were comparable in demographic and baseline clinical variables. The duration of surgery and anesthesia, as well as length of stay in hospital did not differ among the four groups.

**Table 1:** Baseline and perioperative data.

Variable	Group S (*n* = 24)	Group SL (*n* = 24)	Group P (*n* = 25)	Group PL (*n* = 25)	*P*–value
Age, y	50.3 ± 9.3	53.6 ± 10.5	51.1 ± 7.8	50.5 ± 8.1	0.557
BMI, *n* (%)					0.795
≤ 24 kg/m^2^	14 (58.3)	17 (70.8)	16 (64)	13 (52)	
> 24 kg/m^2^	10 (41.7)	7 (29.2)	9 (36)	12 (48)	
ASA, *n* (%)					0.667
ASA I	8 (33.3)	7 (29.2)	5 (20)	6 (24)	
ASA II	16 (66.7)	17 (70.8)	20 (80)	19 (76)	
Duration of surgery, min	82.6 ± 20.4	84.4 ± 21.7	76.8 ± 28.5	77.6 ± 26.6	0.561
Duration of anesthesia, min	104.9 ± 24.8	105.1 ± 24.05	96.1 ± 30.6	97.7 ± 30.5	0.548
Length of stay in hospital, d	5.3 ± 1.8	5.2 ± 1.9	5.9 ± 2.9	5.2 ± 2.2	0.645

Values are shown as mean ± SD or median (IQR) or number (%).

BMI, body mass index; ASA, American Society of Anesthesiologists; Group S, sevoflurane group; Group SL, sevoflurane-lidocaine group; Group P, propofol group; Group PL, propofol-lidocaine group.

### 3.2 Pain scores and analgesics

**Figure 2:**
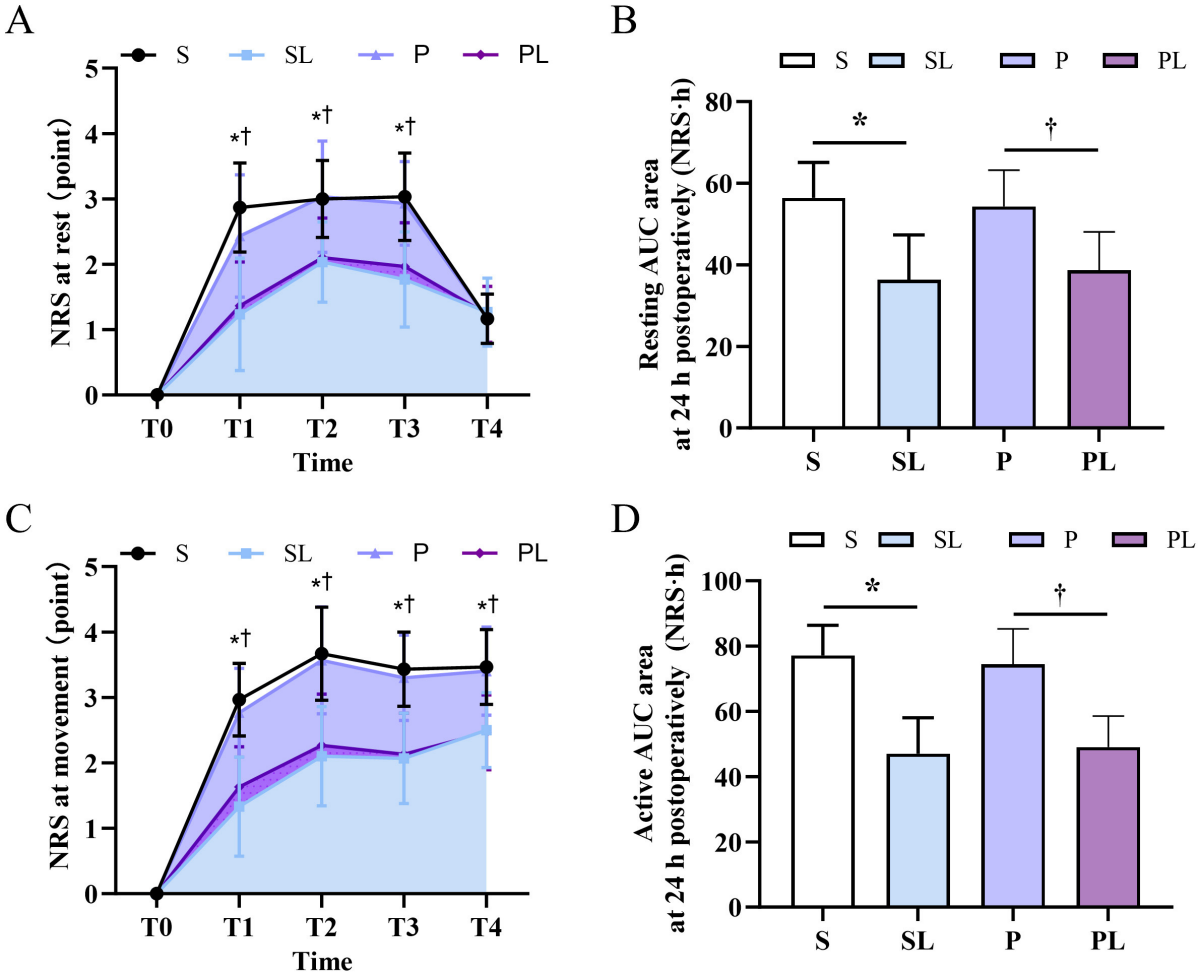
Comparisons of the overall postoperative analgesic efficacy. NRS scores at rest **(A)** and movement **(C)**, and area under the curve **(B, D)**. Groups: S, sevoflurane group; SL, sevoflurane-lidocaine group; P, propofol group; PL, propofol-lidocaine group. Data are presented as median (IQR). Time points: T0 (0h), T1 (3h), T2 (6h), T3 (12h), T4 (24h), postoperatively. *P < 0.001 S vs. SL group; ^†^*P* < 0.001 P vs. PL group.

As shown in Table 2, in the MRM population, the resting and active AUC for NRS score at 24 h after surgery was significantly lower in the lidocaine groups (SL and PL) compared with the non-lidocaine groups (S and P). The Kruskal-Wallis test revealed a significant difference in resting AUC among the four groups (H = 60.0, *P* < 0.001). The Wilcoxon signed-rank test showed that postoperative 24-h resting pain score AUC: median (IQR), SL vs. S group, [37.5 (26.3, 46.9)] vs. [55.5 (51, 64.5)], *P* < 0.0001, PL vs. P group, [37.5 (30.38, 45)] vs. [54 (49.5, 60)], *P* < 0.0001 (Figure 2B, Table 2); The Kruskal-Wallis test revealed a significant difference in active AUC among the four groups (H = 79.6, *P* < 0.001). The Wilcoxon signed-rank test showed that postoperative 24-h active pain score AUC: median (IQR), SL vs. S group, [48 (37.5, 55.5)] vs. [78 (67.5, 84.4)], *P* < 0.0001, PL vs. P group, [48.8 (43.1, 55.5)] vs. [74.3 (68.6, 81.4)], *P* < 0.0001 (Figure 2D, Table 2). No significant differences were observed either in resting or active AUC 24 h postoperatively between the sevoflurane groups (S and SL) and the propofol groups (P and PL) (all *P* > 0.05). Specifically, for resting pain AUC: S vs. P, *P* =0.4189, SL vs. PL, *P* =0.4353 (Figure 2B, Table 2); For active pain AUC: S vs. P, *P* =0.3623, SL vs. PL, *P* =0.4866 (Figure 2D, Table 2).

**Table 2:** Pain scores and analgesics.

Variable	Group S (*n* = 24)	Group SL (*n* = 24)	Group P (*n* = 25)	Group PL (*n* = 25)	*P*–value
Primary outcome (the AUC at 24 h postoperatively)					
Resting AUC (NRS·h)	55.5 (51, 64.5)	37.5 (26.3, 46.9)	54 (49.5, 60)	37.5 (30.38, 45)	<0.0001^*^†
Active AUC (NRS·h)	78 (67.5, 84.4)	48 (37.5, 55.5)	74.3 (68.6, 81.4)	48.8 (43.1, 55.5)	<0.0001^*^†
Secondary outcomes					
Pain scores–Time					
Resting NRS– Pre–op	0	0	0	0	1
Resting NRS−0 post–op	0	0	0	0	1
Resting NRS−3h post–op	3 (3, 3)	1 (1, 2)	3 (2, 3)	1 (1, 2)	<0.0001^*^†
Resting NRS−6h post–op	3 (3, 3)	2 (2, 2)	3 (3, 3.25)	2 (2, 2.25)	<0.0001^*^†
Resting NRS−12h post–op	3 (3, 3.25)	2 (1, 2)	3 (2.75, 3)	2 (1.75, 2)	<0.0001^*^†
Resting NRS−24h post–op	1 (1, 1)	1 (1, 1.25)	1 (1, 1)	1 (1, 1.25)	0.826
Active NRS–Pre–op	0	0	0	0	1
Resting NRS−0 post–op	0	0	0	0	1
Active NRS−3h post–op	3 (3, 3)	1 (1, 2)	3 (2, 3)	2 (1, 2)	<0.0001^*^†
Active NRS−6h post–op	4 (3, 4)	2 (2, 3)	4 (3, 4)	2 (2, 3)	<0.0001^*^†
Active NRS−12h post–op	3 (3, 4)	2 (2, 3)	3 (3, 4)	2 (2, 3)	<0.0001^*^†
Active NRS−24h post–op	3.5 (3, 4)	2.5 (3, 4)	3.5 (3, 4)	2.5 (2, 3)	<0.0001^*^†
Opioid consumption					
Sufentanil, μg	18.2 ± 2.9	17.8 ± 1.9	18.2 ± 2.2	18.2 ± 1.9	0.903
Remifentanil, mg	1.0 ± 0.3	1.0 ± 0.3	1.1 ± 0.5	1.0 ± 0.4	0.741
Etomidate, mg	18.2 ± 2.9	17.8 ± 1.9	18.2 ± 2.2	18.2 ± 1.9	0.903
Propofol, mg	–	–	215.2 ± 53.4	231 ± 46.1	0.268
Rocuronium, mg	43.8 ± 7.4	40.1 ± 6.5	41.5 ± 7.0	39.7 ± 6.4	0.156
Lidocaine/Normal saline, ml	22.8 ± 5.5	23.6 ± 3.6	20.7 ± 6.3	21.9 ± 5.7	0.275
Ephedrine, mg	6.5 ± 2.2	6.0 ± 2.9	5.8 ± 2.7	6.4 ± 3.1	0.786
Postoperative rescue analgesia frequency	2 (8.3)	2 (8.3)	2 (8)	2 (8)	0.994
Ketorolac, mg	0 (0, 0)	0 (0, 0)	0 (0, 0)	0 (0, 0)	0.955
Paracetamol, g	0	0	0	0	1
Patient satisfaction	8.67 ± 0.9	9 ± 0.8	8.8 ± 0.9	9.1 ± 0.6	0.246

Values are shown as mean ± SD or median (IQR) or number (%).

AUC, the total area under the pain curve; NRS, digital rating pain scores; Pre-op, pre-operation; post-op, post-operation; Group S, sevoflurane group; Group SL, sevoflurane-lidocaine group; Group P, propofol group; Group PL, propofol-lidocaine group.

^*^P < 0.001 S vs. SL group; ^†^*P* < 0.001 P vs. PL group.

As shown in Table 2 and Figure 2, the Friedman test indicated no significant differences in resting and active NRS pain scores among the four groups at preoperative and 0 h postoperatively (H = 0, *P* = 1). In contrast, significant differences were observed among the four groups at 3, 6, and 12 h postoperatively for both resting and active NRS scores (all *P* < 0.001). The Wilcoxon signed-rank test demonstrated that, at 3, 6, and 12 h postoperatively, both resting and active NRS pain scores were significantly lower in the lidocaine groups (SL and PL) than in the non-lidocaine groups (S and P) (S vs. SL and P vs. PL, both *P* < 0.001). Conversely, no significant differences in pain scores were detected between the sevoflurane and propofol groups at these time points (S vs. P, SL vs. PL, all *P* > 0.05). At 24 h postoperatively, no significant difference was found among the four groups in the resting NRS scores (H = 0.899, *P* = 0.826), whereas a significant difference was observed in the active NRS scores. Specifically, the active NRS pain scores were significantly lower in the lidocaine groups (SL and PL) than in the non-lidocaine groups (S and P) (S vs. SL and P vs. PL, both *P* < 0.001), while no significant differences were observed between the sevoflurane and propofol groups (S vs. P and SL vs. PL, all *P* > 0.05). Additionally, one-way ANOVA revealed no statistically significant differences among the four groups in opioid consumption, doses of anesthetics and analgesics, or patient satisfaction (Table 2).

Rescue analgesic use: During the study period, no patients received oral acetaminophen. And 8 patients (2 per group) received a single intramuscular dose of ketorolac (30 mg) because they had an NRS pain score ≥ 4, and each of these patients received a total dose of 30 mg. The Kruskal–Wallis test revealed no significant differences among the four groups in the rate of analgesic use or the dosage administered (*P* > 0.05). The remaining patients did not receive any rescue analgesics (Table 2).

### 3.3 Immune cells and inflammatory factors

**Figure 3:**
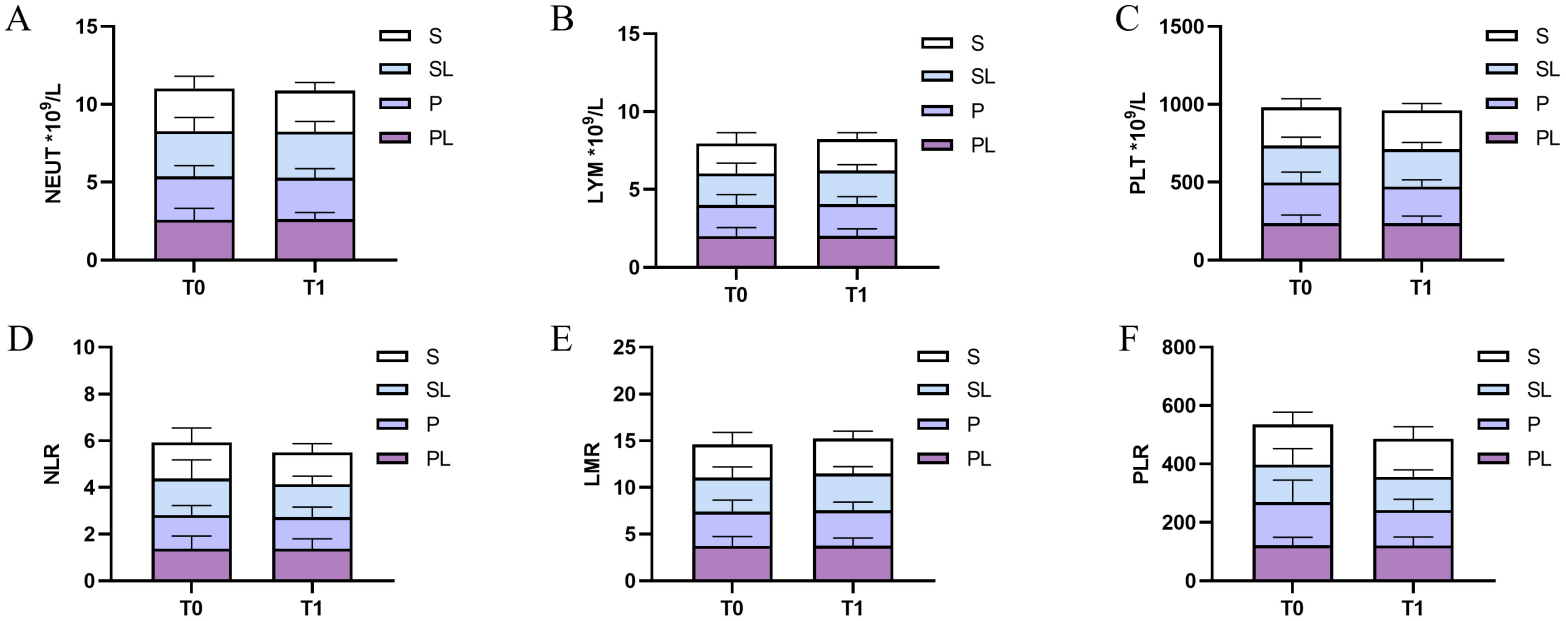
Comparisons of the immune cells. The system immune cell variables include NEUT **(A)**, LYM **(B)**, PLT **(C)**, NLR **(D)**, LMR **(E)**, PLR **(F)**. Data are presented as the median (IQR). S, sevoflurane group; SL, sevoflurane-lidocaine group; P, propofol group; PL, propofol-lidocaine group. NEUT, absolute neutrophil counts; LYM, absolute lymphocyte counts; PLT, platelet counts; NLR, neutrophil-to-lymphocyte ratio; LMR, lymphocyte-to-monocyte ratio; PLR, platelet-to-lymphocyte ratio. Time points: T0 (preoperative), T1 (3h postoperatively).

The Kruskal-Wallis test revealed that there is no significant difference in the Immune cells outcomes among the four groups before and 3 h after surgery, including absolute counts of neutrophils, lymphocytes, and platelets, and NLR, LMR, and PLR (all *P* > 0.05; Figure 3).

**Figure 4:**
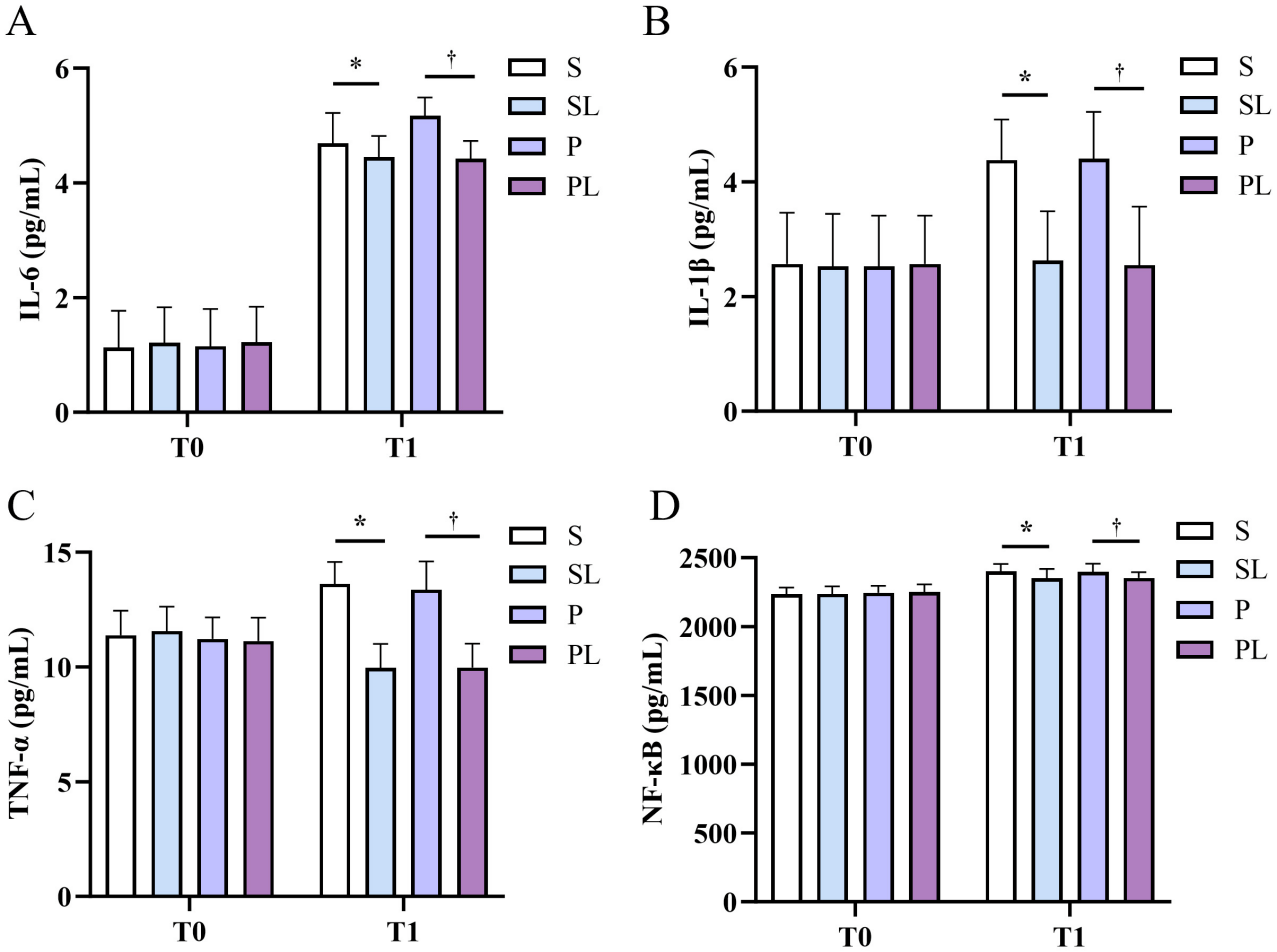
Comparisons of the inflammatory factors. The inflammatory factors variables include IL-6 **(A)**, IL-1β **(B)**, TNF-α **(C)**, NF-κB0 **(D)**. Data are presented as the median (IQR). S, sevoflurane group; SL, sevoflurane-lidocaine group; P, propofol group; PL, propofol-lidocaine group. Time points: T0 (preoperative), T1 (3h postoperatively). *P < 0.001 S vs. SL group; ^†^*P* < 0.001 P vs. PL group.

As shown in Figure 4, the Kruskal-Wallis test revealed that there is no showed no significant difference in the serum levels of inflammatory cytokines (IL-6, IL-1β, TNF-α, NF-κB) among the four groups before surgery (all *P* > 0.05). At 3 h postoperatively, the serum levels of IL-6, IL-1β, TNF-α, NF-κB were significantly lower in the lidocaine groups (SL and PL) compared respectively with the non-lidocaine groups (S and P) (S vs. SL, P vs. PL, both *P* < 0.001). However, no significant differences were observed in these inflammatory cytokines between the sevoflurane groups and the propofol groups (S vs. P, SL vs. PL, all *P* > 0.05). (Figure 4).

### 3.4 Perioperative adverse reactions

No adverse events of respiratory depression, local anesthetic systemic toxicity (LAST), or allergic reactions were observed. Neurological symptoms, including perioral paresthesia, tinnitus, dizziness, blurred vision, and muscle twitching, were also absent. And the Kruskal-Wallis test revealed that hypertension, hypotension, nausea, vomiting, and arrhythmia were reported in all four groups, with no significant differences in their incidences among the groups (all *P* > 0.05; Table 3).

**Table 3:** Perioperative adverse reactions.

Variable	Group S (*n* = 24)	Group SL (*n* = 24)	Group P (*n* = 25)	Group PL (*n* = 25)	*P–*value
PONV	3 (12.5)	2 (8.3)	2 (8)	3 (12)	0.933
Respiratory depression	0	0	0	0	1
Hypotension	2 (8.3)	3 (12.5)	2 (8)	2 (8)	0.771
Hypertension	5 (20.8)	4 (16.7)	5 (20)	4 (16)	0.967
Arrhythmia	2 (8.3)	1 (4.2)	1 (4)	1 (4)	0.876
Neurological symptoms	0	0	0	0	1
LAST	0	0	0	0	1
Anaphylaxis	0	0	0	0	1

Values are shown as number (%).

PONV, postoperative nausea, and vomiting; neurological symptoms: perioral numbness, tinnitus, dizziness, visual disturbances, muscle twitching; LAST, local anesthetic systemic toxicity; Group S, sevoflurane group; Group SL, sevoflurane-lidocaine group; Group P, propofol group; Group PL, propofol-lidocaine group.

## 4 Discussion

In this study, we estimated the effects of intraoperative intravenous lidocaine on acute postoperative pain, in patients undergoing MRM under propofol or sevoflurane maintenance anesthesia. According to the results, lidocaine infusion significantly reduced the resting and active AUC under the pain score curve at 24 h after surgery and resulted in lower NRS scores at 3, 6, and 12 h postoperatively. These benefits were independent of the maintenance anesthetic used (sevoflurane vs. propofol). Our study reaffirms the feasibility of lidocaine as an anesthetic adjunct and confirms its clinical value in multimodal analgesic protocols.

Optimizing perioperative pain management is critical for improving patient comfort, reducing complication rates, and enhancing postoperative recovery (25). The AUC of postoperative pain scores serves as a robust metric for assessing analgesic efficacy, as it reflects cumulative pain burden over 24 h and minimizes bias associated with single-timepoint assessments (26). A reduced AUC indicates superior pain control and greater effectiveness of the analgesic strategy (27).

In our study, lidocaine significantly decreased the 24-h resting and active AUC for NRS pain score in patients undergoing MRM. And secondary outcomes revealed that lidocaine-treated patients exhibited lower resting and active NRS scores at 3, 6, and 12 h postoperatively compared with controls. These findings are consistent with previous reports (28–31). Klinger et al. demonstrated that intravenous lidocaine significantly reduced 24-h VAS pain scores following cardiac surgery (28). A meta-analysis by Weibel et al. confirmed that perioperative intravenous lidocaine significantly decreases postoperative pain scores in abdominal surgery (29). And Popa et al. reported that lidocaine improves early recovery quality in breast surgery (30). Furthermore, a systematic review and meta-analysis by Hussain et al., which included 13 randomized controlled trials involving 1,039 patients undergoing breast cancer surgery, showed that perioperative intravenous lidocaine significantly reduced resting pain scores at 1, 6, 12, and 24 h postoperatively and decreased opioid consumption within the first 24 h after surgery (31). Collectively, these evidence supports the clinical value of lidocaine for perioperative analgesia in breast surgery. Given that AUC was not pre-specified, all analyses related to AUC should be interpreted as exploratory, and the findings require confirmation in prospectively designed trials.

The observed clinical effects are supported by well-characterized pharmacological mechanisms (32–35). Intravenous lidocaine exerts multimodal analgesia via actions at peripheral, immune, and central nervous system sites (32). Peripherally, it blocks voltage-gated sodium channels on injured nerve terminals, suppressing ectopic discharges and reducing aberrant nociceptive input (33). At the immune level, lidocaine attenuates neurogenic inflammation (34); Our research findings indicates that lidocaine exerts anti-inflammatory effects by regulating immune cell function and inhibiting the release of pro-inflammatory cytokines, rather than altering the number and proportion of immune cells. Centrally, lidocaine inhibits NMDA receptors and voltage-gated sodium and potassium channels in spinal dorsal horn neurons, thereby suppressing central sensitization and nociceptive amplification (35). These integrated mechanisms constitute the pharmacological basis for effective analgesia of lidocaine.

Despite the overall analgesic benefits, certain findings diverged from previous reports. Contrary to the observations of Hussain et al. (31), in our study, there was no significant inter group difference in resting or active pain scores among the four groups at 24-h postoperatively. The inconsistent results of these studies could be attributed to differences in procedures, concentrations of local anesthetic, single injection versus infusion, and a multi-modal analgesic regimen. Furthermore, although studies have reported that lidocaine reduces perioperative opioid consumption (30, 36), we did not observe inter group differences in anesthesia or analgesic drug doses, which may be attributed to differences in surgical types and tissue trauma levels that affect baseline pain intensity, as well as changes in lidocaine administration regimens (such as push dose and infusion rate). And previous meta-analyses (37) have suggested that perioperative intravenous lidocaine shortens hospital stay and improves patient satisfaction. However, these benefits were not replicated in our study. This discrepancy may reflect insufficient statistical power for the secondary outcomes (e.g., NRS, medication dose, length of hospital stay), as the sample size calculation in this study was based on the primary endpoint of the AUC for postoperative pain scores. Therefore, secondary outcome results should be interpreted cautiously, and further validation in larger-sample studies is warranted. Additionally, standardized enhanced recovery protocols may have attenuated the independent effect of lidocaine on overall hospitalization duration.

Current evidence regarding the comparative effects of propofol and sevoflurane on postoperative pain remains inconclusive. Some studies have reported that propofol reduces postoperative pain compared with sevoflurane (17, 18); however, recent meta-analyses have demonstrated no significant difference between the two agents in terms of postoperative pain outcomes (19, 20). In the present study, no significant differences in pain scores were observed between the propofol and sevoflurane groups, a finding consistent with Choi et al. (38). Propofol possesses anti-inflammatory and antioxidant properties (39), while sevoflurane exerts neuroregulatory effects through multiple targets (40); both could influence the analgesic efficacy of lidocaine theoretically. Nevertheless, in our study, no significant difference was found in the analgesic effect of lidocaine between two different anesthesia maintenance drugs (propofol and sevoflurane), indicating that its adjuvant analgesic effect has a certain degree of universal adaptability.

After determining that the analgesic effect of lidocaine is independent of anesthesia techniques, we further evaluated its clinical safety. Safety assessments revealed no life-threatening adverse events attributable to lidocaine in this study. The incidence of postoperative nausea and vomiting, hypertension, hypotension, and arrhythmias did not differ significantly among the four groups. These findings further corroborate the favorable safety profile of perioperative intravenous lidocaine, consistent with previous reports (41).

This study found that perioperative intravenous lidocaine significantly reduced the resting and active AUC of the NRS score within 24 h after surgery in MRM patients. However, it did not significantly reduce total opioid consumption, shorten hospital stay, or alter the incidence of postoperative adverse events. This contradictory result carries important clinical implications. First, the resting and active AUC of the NRS score is a composite measure of a patient's overall pain experience; its reduction suggests that lidocaine alleviates the total burden and variability of postoperative pain. Second, lidocaine achieves a lower resting and active AUC of the NRS score under the same level of opioid consumption, avoiding the side effects (such as nausea, vomiting and excessive sedation) caused by the additional administration of opioids to reduce pain scores. Therefore, despite not reducing opioid use, lidocaine still offers clinical value by improving the quality of pain control. Future studies should focus on early recovery parameters (acute pain, early ambulation, and sleep quality) and the incidence of chronic pain to further validate its long-term benefits.

Our anesthetic protocol, while necessary for the study design, contains several elements that merit critical discussion. First, the use of etomidate for induction, even as a single dose, may suppress cortisol synthesis for more than 24 h, potentially affecting postoperative stress response and pain perception (42). Second, the combination of sufentanil (induction) and remifentanil (maintenance) involves two different opioids. Although no statistically significant differences in remifentanil infusion dose were observed among the groups, the potential influence of intraindividual infusion variability on hyperalgesia cannot be completely excluded (43). Third, the use of a laryngeal mask airway together with a neuromuscular blocker remains controversial and is not universally accepted; it was employed here to ensure stable ventilation and optimal surgical conditions, but poses risks of gastric regurgitation and mask leakage. This study was conducted under strict fasting and monitoring conditions, and all procedures were performed by experienced anesthesiologists. Readers are advised to interpret our findings with these limitations in mind, and the protocol should not be extrapolated to routine clinical practice without further validation. And the preoperative and 3-h postoperative inflammatory measures cannot reflect the complete dynamic trajectory of inflammatory markers. Future studies should include more time points to dynamically monitor the impact of lidocaine on inflammatory indicators. Besides, this study did not employ a standardized postoperative analgesia protocol, which poses a limitation to the external validity of the conclusions. The specific role of lidocaine in the setting of standardized analgesia remains to be elucidated. Future studies should be conducted within a more standardized multimodal analgesic regimen to validate these findings.

In conclusion, perioperative intravenous lidocaine effectively reduces early postoperative pain scores and cumulative pain burden in patients undergoing MRM. The analgesic effects of lidocaine are mediated by integrated mechanisms involving the peripheral, immune, and central nervous systems. Although no significant advantages were observed in secondary outcomes such as hospital stay, satisfaction, or opioid consumption, possibly due to sample size limitations and standardized perioperative protocols, the consistent analgesic efficacy and favorable safety profile support lidocaine as a valuable adjunct in multimodal analgesic regimens. Future studies should optimize administration protocols and validate these findings across larger samples and diverse surgical types.

### 4.1 Limitations

This study has several limitations. First, the relatively small sample size may have limited the statistical power to detect subtle differences among groups. Second, plasma lidocaine concentrations were not measured in this study. Although our infusion protocol was based on previous clinical studies that demonstrated safety and efficacy, and no lidocaine-related toxicity was observed during rigorous intraoperative monitoring, the lack of plasma concentration data remains a major limitation. Future studies are warranted to measure plasma lidocaine concentrations in order to more precisely define the pharmacokinetic-pharmacodynamic relationship and safety margin of lidocaine. Third, the observation period was restricted to the acute postoperative phase (24 h after surgery), and long-term follow-up for chronic postsurgical pain was not conducted. Future studies with larger sample sizes and extended follow-up durations are warranted to validate these findings.

## 5 Conclusion

In this prospective study, intraoperative intravenous lidocaine was associated with reduced early postoperative inflammation and improved analgesic outcomes. These benefits were independent of the maintenance anesthetic used (sevoflurane and propofol). These findings suggest that intravenous lidocaine may serve as a safe and effective adjunct in multimodal perioperative analgesia. However, given the multiple confounding factors that may affect the results of this study due to its design, further randomized controlled trials are required to confirm causality and assess long-term clinical and mechanistic outcomes.

## Data Availability

The original contributions presented in the study are included in the article/Supplementary material, further inquiries can be directed to the corresponding authors.
